# Intravitreal Dexamethasone in the Management of Delayed-Onset Bleb-Associated Endophthalmitis

**DOI:** 10.1155/2012/503912

**Published:** 2012-01-11

**Authors:** David J. Jacobs, Avinash Pathengay, Harry W. Flynn, Theodore Leng, Darlene Miller, Wei Shi

**Affiliations:** ^1^Department of Ophthalmology, Bascom Palmer Eye Institute, Miller School of Medicine, University of Miami, Miami, FL 33136, USA; ^2^Department of Ophthalmology, Byers Eye Institute at Stanford, Stanford School of Medicine, Palo Alto, CA 94303, USA

## Abstract

*Purpose.* To report the visual acuity (VA) outcomes and culture results of delayed-onset bleb-associated endophthalmitis (BAE) with and without intravitreal dexamethasone (IVD). *Methods.* Retrospective nonrandomized comparative case series of BAE at Bascom Palmer Eye Institute between January 1, 1996 and December 31, 2009. Clinical data were compared using the 2-sided Student's *t*-test for patients who received IVD and patients who did not receive IVD. *Results.* 70/83 (84%) received IVD, and 13/83 (16%) did not receive IVD. Mean baseline VA was 20/90 in the IVD group and 20/70 in the group that did not receive IVD (*P* = 0.57). Mean presenting VA was 0.9/200 in the IVD group and 1.7/200 in the group that did not receive IVD (*P* = 0.23). Repeat cultures were positive in 2/70 (3%) IVD cases and 1/13 (8%) cases that did not receive IVD (*P* = 0.57). Mean VA at 1 month was 5/200 in the IVD group and 1.8/200 in the group that did not receive IVD, logMARΔ of 0.85 and 1.56, respectively (*P* = 0.02). Mean VA at 3 months was 7/200 in the IVD group and 3/200 in the group that did not receive IVD, logMARΔ of 0.74 and 1.33, respectively (*P* = 0.14). *Conclusion.* In the current study of BAE, IVD was associated with improved short-term VA outcomes without an increased rate of persistent infection.

## 1. Introduction

Since 1974 intravitreal dexamethasone (IVD) has been used as an adjunct to intravitreal antibiotics in the management of bacterial endophthalmitis [1–3]. In 1992, Irvine et al. reported favorable outcomes in a series of Gram-negative endophthalmitis cases treated with adjunctive IVD [2]. In 2004 43% of retina specialists responded that they use IVD in the management of postcataract endophthalmitis [4]. The role of IVD in the management of bacterial endophthalmitis remains controversial due to contradictory results reported by small, comparative studies [5–8].

In delayed-onset bleb-associated endophthalmitis (BAE), the majority of reported cases, 53–82%, received adjunctive IVD [9–12]. No BAE series however has yet reported the VA outcomes of cases treated with and without IVD. Unlike postcataract endophthalmitis, BAE is commonly associated with virulent *Streptococcus* and Gram-negative organisms [10–13]. If IVD potentiates intraocular infection then VA outcomes may be worse in BAE cases treated with IVD. Additionally, the culture data of second biopsies in BAE cases treated with IVD may manifest a higher rate of persistent infection. The current study reports the VA outcomes and culture results of BAE cases treated with and without IVD to further clarify the role IVD plays in the management of bacterial endophthalmitis.

## 2. Methods

The study protocol was approved by the Institutional Review Board of the University of Miami Miller School of Medicine Subcommittee for the Protection of Human Subjects in Research. The medical records and microbiologic records of all patients treated for BAE at Bascom Palmer Eye Institute (BPEI) between January 1, 1996 and December 31, 2009 were reviewed. As a nonrandomized comparative case series the decision to use or not use IVD was made by the individual treating physician and did not involve a prospective protocol. All patients had prior glaucoma filtering surgery. BAE was defined as intraocular infection with vitreous involvement receiving treatment with intravitreal antibiotics. Patients with tube shunts as the filtering mechanism, bleb infection only (no posterior inflammation), onset within 1 month of glaucoma surgery, and inadvertent filtering blebs after cataract surgery were excluded. Clinical history and presentation, treatment, intraocular culture data, VA outcomes, and factors affecting VA were recorded. The current study included clinical information from the BPEI series of BAE previously published [9, 13].

Snellen VAs were converted to logMAR equivalents for statistical analysis; VAs of HM, LP, and NLP were assigned logMAR values of 2.6, 3, and 4 respectively. Change in VA was determined by comparing the last recorded VA before the onset of endophthalmitis (pre-endophthalmitis VA) with VA at 1 and 3 months. Three or more lines of improvement (≥3 lines improvement) were determined by comparing the VA at presentation of endophthalmitis (presentation VA) with the VA at 1 and 3 months. The mean logMAR change after presentation (logMARΔ) and other clinical data were grouped according to IVD use and compared using the 2-sided Student's *t*-test. Logistic regression was used to determine the odds ratio for IVD as a predictive factor for ≥3 lines improvement. A *P* value of ≤0.05 was considered statistically significant.

## 3. Results

In the current study, 86 eyes were identified. Excluded were 1 eye with a preexisting tube shunt and 2 eyes that underwent primary evisceration. Of the 83 eyes, 70 (84%) received IVD and 13 (16%) did not receive IVD. None of the patients received systemic steroid. In all cases, the causative organisms were sensitive to the intravitreal antibiotics clinically administered. Baseline demographics, clinical presentation, and initial culture results were similar between the two groups with a few exceptions (Table 1).

A greater percentage of IVD cases presented with poor view of the fundus, 69% compared to 39%. The majority of both groups received an initial treatment of tap and injection (T&I); however, a higher percentage received pars plana vitrectomy (PPV) in the IVD group, 41% compared to 8%. Also a higher percentage of IVD cases were culture-positive, 66% compared to 46%, but this difference did not reach significance.

Repeat cultures were performed during a second procedure in 11/70 (16%) IVD cases and 3/13 (23%) of cases that did not receive IVD (Table 2).There was no significant difference in primary or repeat culture-positive results between the two groups. The repeat culture-positive rate was 2/70 (3%) for IVD cases and 1/13 (8%) for the cases that did not receive IVD. In each of these 3 cases the same causative organism was isolated in the repeat culture as in the initial culture. Repeat cultures were positive in 1/21 (5%) *Streptococcus* and 2/6 (33%) *Enterococcus* cases.

IVD cases had worse mean pre-endophthalmitis and presentation VA but this did not reach significance (Table 3).

At 1 month mean VA was 5/200 in the IVD group and 1.8/200 in the group that did not receive IVD, logMARΔ of 0.85 and 1.56, respectively (*P* = 0.02). At 3 months mean VA was 7/200 in the IVD group and 3/200 in the group that did not receive IVD, logMARΔ of 0.74 and 1.33, respectively (*P* = 0.14). A higher percentage of IVD cases achieved ≥3 lines improvement at 1 and 3 months. Logistic regression showed that IVD was a significant predictive factor of ≥3 lines improvement at both 1 and 3 months (Table 4).

## 4. Discussion

**Figure 1:**
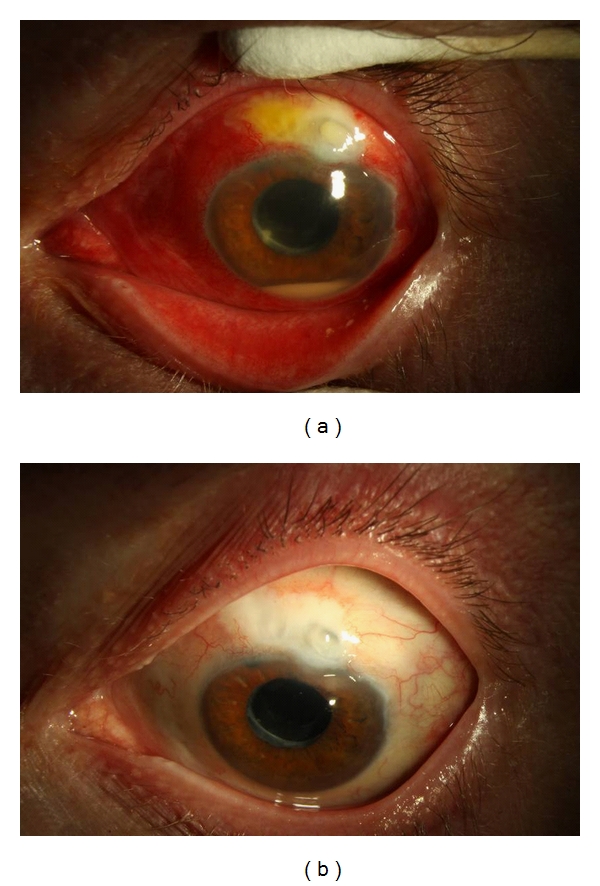
Photographs of the left eye of 55-year-old male presenting with BAE from *Moraxella*. (a) Presenting VA: HM, IOP: 19 mmHg. Treatment: pars plana vitrectomy with intravitreal Vancomycin, Ceftazidime, and Dexamethasone. (b) At 3 months VA: 20/40, IOP: 14 mmHg.

Corticosteroids are often used as an important adjunct to antibiotics and PPV in the management of infectious bacterial endophthalmitis (Figure 1). Corticosteroids are known to reduce the degree of inflammation caused by toxins liberated from microorganisms. The role of IVD in the management of postcataract bacterial endophthalmitis is unclear due to contradictory results of small, comparative studies (Table 5).

Das et al. reported favorable results of reduced intraocular inflammation at 1 and 4 weeks in eyes treated with IVD [5]. Gan et al. additionally found a trend toward better visual outcomes at 3 and 12 months in eyes treated with IVD [6]. In contrast, Hall et al. reported no difference in inflammation and VA outcomes at last followup in eyes treated with IVD [7]. Shah et al. found worse VA outcomes at 1, 3, and 6 months in eyes treated with IVD [8]. The present series was unique as it was the first to study BAE cases that had a higher rate of *Streptococcus* and Gram-negative cases.

BAE studies are limited by the relatively small number of BAE cases. Conclusions in BAE studies are found in the inherent limitations of retrospective nonrandomized data. The majority of cases in the present study received IVD which is similar to other BAE series [9–12]. Overall the two groups compared in this study had similar baseline demographic, clinical presentation, and initial culture data (Table 1).

A difference was found in the initial treatment of the two groups. The majority of cases in both groups received T&I as the initial treatment, however the percentage that received initial PPV was higher in the IVD group. The effect this difference had on the VA outcomes is unclear. A comparison of PPV and T&I cases in this series showed that PPV cases had significantly worse mean VA at presentation and 3 months (Table 6).

When presentation of VA was LP or worse, 3-month-logMARΔ was worse in the PPV group but not significantly (Table 7).

As VA outcomes with PPV were worse than T&I in this series, it is unlikely that improved VA outcomes in the IVD group were due to a higher percentage of initial treatment with PPV.

The repeat culture results in this series were similar to the Endophthalmitis Vitrectomy Study (EVS). In the EVS, 14 of 420 (3.3%) had positive repeat cultures [14]. In the present series the overall repeat culture-positive rate was 3 of 83 (3.6%). Of note the EVS did not use IVD and the EVS had a lower rate of *Streptococcus* and Gram-negative organisms, yet the rate of persistent infection was similar in the EVS to the present study. The present study confirms the observation made by Shaarawy et al. that persistent infection can occur in bacterial endophthalmitis and appears to be more common with virulent organisms such as *Streptococcus* and *Enterococcus *[15]. Although persistent infection occurred in the present BAE series there was not a higher rate among the cases treated with IVD.

VA outcomes in the present series confirm the clinical observation by Irvine et al. that intraocular steroids appeared to hasten visual recovery [2]. At 1 month, 67% gained ≥3 lines in the IVD group compared to 25% in the group that did not receive IVD. The VA gains in the IVD group were more significant at 1 month than 3 months. Logistic regression did show that IVD was a predictive factor of ≥3 lines of improvement at both 1 and 3 months. VA gains may have been due to a decrease in intraocular inflammation, but a standardized manner of grading inflammation was not employed in the present series.

Limitations of the current study include the retrospective nature, small sample size in the control arm, and lack of a definitive treatment protocol. This study does demonstrate that IVD was associated with improved short-term VA outcomes and did not potentiate infection in BAE.

## Figures and Tables

**Table 1:** Baseline demographics, clinical presentation, and initial culture results.

	IVD	No IVD	*P* value
	70/83 (84%)	13/83 (16%)	
Age			
Mean, SD	74 yr (12)	70 yr (14)	0.27
Gender			
Female	34 (49%)	8 (62%)	0.39
Male	36 (51%)	5 (39%)	
Diabetes mellitus			
Present	9 (13%)	2 (15%)	0.81
Absent	61 (87%)	11 (85%)	
Antimetabolites (MMC or 5FU)			
Used	45 (64%)	7 (54%)	0.47
Not used	25 (36%)	6 (46%)	
Mean time of onset, SD	60 mo (43)	49 mo (55)	0.46
Bleb leak			
Present	16 (23%)	5 (38%)	0.23
Absent	54 (77%)	8 (62%)	
Anterior chamber			
Hypopyon	48 (69%)	10 (77%)	0.55
View to fundus			
Hazy	22 (31%)	8 (62%)	0.04
Poor/none	48 (69%)	5 (39%)	
Intraocular Pressure			
Presentation, SD	20 (14)	19 (12)	0.8
Treatment, initial			
Tap and injection	41 (59%)	12 (92%)	0.03
Pars plana vitrectomy	29 (41%)	1 (8%)	
Treatment, additional			
Filtering procedure	12 (17%)	1 (8%)	0.39
Pars plana vitrectomy	21 (30%)	2 (15%)	0.28
Culture results			
Culture positive	46 (66%)	6 (46%)	0.18
Culture negative	24 (34%)	7 (54%)	
Gram-positive cases	33 (47%)	4 (31%)	0.28
*Streptococcus *	19 (27%)	2 (15%)	0.37
Coagulase-negative *Staphylococcus *	7 (10%)	2 (15%)	0.57
*Enterococcus *	6 (9%)	0	0.27
*Staphylococcus aureus *	1 (1%)	0	0.67
Gram-negative cases	12 (17%)	2 (15%)	0.88
*Moraxella *	8 (11%)	0	0.2
*Pseudomonas *	2 (3%)	1 (8%)	0.39
*Serratia *	1 (1%)	1 (8%)	0.18

**Table 2:** Repeat culture results.

	IVD	No IVD	*P* value
	70/83 (84%)	13/83 (16%)	
Repeat Cultures Performed:			
Number of eyes	11 (16%)	3 (23%)	0.52
Mean time, days (range)	20 (1–60)	14 (2–30)	0.64
Primary culture results			
*Streptococcus *	4 (36%)	2 (67%)	
*Enterococcus faecalis *	2 (18%)	0	
Coagulase-neg *Staph. *	2 (18%)	1 (33%)	
*Enterobacter aerogenes *	1 (9%)	0	
No growth	2 (18%)	0	0.43
Repeat culture results			
*Streptococcus *	0	1 (33%)	
*Enterococcus faecalis *	2 (18%)	0	
Coagulase-neg *Staph. *	0	0	
*Enterobacter aerogenes *	0	0	
No growth	9 (82%)	2 (67%)	0.57

Repeat culture positive rate	2 (3%)	1 (8%)	0.57

**Table 3:** VA outcomes.

	IVD	No IVD	*P* value
	71/84 (84%)	13/84 (16%)	
VA, pre-endophthalmitis	*n* = 67	*n* = 13	
Mean	20/90	20/70	0.57
Range	20/20-LP	20/25–20/400	
VA, presentation	*n* = 70	*n* = 13	
Mean	0.9/200	1.7/200	0.23
Range	20/40-NLP	20/80-LP	
VA, 1 month	*n* = 66	*n* = 12	
Mean	5/200	1.8/200	0.14
Range	20/25-NLP	20/25-NLP	
≥3 lines Improvement	44 (67%)	3 (25%)	0.01
logMARΔ	0.85	1.56	0.02
VA, 3 months	*n* = 56	*n* = 9	
Mean	7/200	3/200	0.36
Range	20/25-NLP	20/25-LP	
≥3 lines Improvement	36 (64%)	3 (33%)	0.14
logMARΔ	0.74	1.33	0.14

**Table 4:** Predictive factor ≥3 lines improvement.

IVD versus No IVD	Odds ratio, (CI)	*P* value
1 month	7.04 (1.63,30.43)	0.01
3 months	5.21 (1.07,25.37)	0.04

**Table 5:** Comparative studies of IVD for bacterial endophthalmitis.

	Clinical setting		Culture results		Inflammation	VA outcomes	Time
	*n*	*Staph epi.*	*Strep/Enterococcus*	Gram-negative			
Das et al. [5]	Postcataract and trauma						
IVD	29	n/a	n/a	n/a	2.6 score	86% success	3 months
No IVD	34				3.2 score^1^	71% success	
Shah et al. [8]	Postcataract						
IVD	26	31%	12%	0	n/a	20/70 median	6 months
No IVD	31	35%	13%	3%		20/50 median^2^	
Gan et al. [6]	Postcataract						
IVD	16	39%	8%	0	n/a	85% 20/200 or better	3 months
No IVD	13	50%	6%	0		50% 20/200 or better^3^	
Hall et al. [7]	Postcataract						
IVD	26	46%	23%	0	0.3 cell/flare	20/40 median	last followup
No IVD	38	37%	5%	0	0.3 cell/flare	20/50 median	
Jacobs et al.	Bleb-associated						
IVD	70	10%	36%	17%	n/a	7/200 mean	3 months
No IVD	13	15%	15%	15%		3/200 mean^4^	

^1^Relative change in inflammation showed statistical significance at 1 and 4 weeks, not at 3 months. ^2^ *P* < 0.05  , ^3^ *P* = 0.055. ^4^Relative logMARΔ showed statistical significance at 1 month, not at 3 months.

**Table 6:** PPV versus T&I in present BAE series.

	PPV	T&I	*P* value
	29/83 (35%)	54/83 (65%)	
VA, pre-endophthalmitis	*n* = 28	*n* = 53	
Mean	20/55	20/50	0.3
Range	20/20-CF	20/20-LP	
VA, presentation	*n* = 30	*n* = 54	
Mean	LP	HM	0.02
Range	20/80-LP	20/40-NLP	
VA, 3 months	*n* = 27	*n* = 39	
Mean	3/200	20/390	
Range	20/25-NLP	20/25-LP	
logMARΔ	1.23	0.57	0.02

**Table 7:** PPV versus T&I: presentation VA of LP or worse in present BAE series.

	PPV	T&I	*P* value
	18/26 (69%)	8/26 (31%)	
VA, pre-endophthalmitis	*n* = 18	*n* = 8	
Mean	20/65	20/270	0.16
Range	20/20–1/200	20/25-HM	
VA, presentation	*n* = 18	*n* = 8	
Mean	LP	LP	0.35
Range	LP	LP-NLP	
VA, 3 months	*n* = 17	*n* = 5	
Mean	1/200	1/200	
Range	20/60-NLP	20/200-LP	
logMARΔ	1.71	1.18	0.46
